# Impact of acetate on CO_2_ fixation pathways in thermophilic and hydrogenotrophic bacteria

**DOI:** 10.1093/ismeco/ycaf227

**Published:** 2025-12-06

**Authors:** Yoko Chiba, Tomomi Sumida, Masafumi Kameya, Yuto Fukuyama, Tomoyuki Wakashima, Shigeru Shimamura, Ryoma Kamikawa, Yoshito Chikaraishi, Takuro Nunoura

**Affiliations:** Center for Sustainable Resource Science, RIKEN, Wako, Saitama 351-0198, Japan; Institute for Extra-cutting-edge Science and Technology Avant-garde Research (X-star) Japan Agency for Marine-Earth Science and Technology (JAMSTEC), Yokosuka, Kanagawa 237-0061, Japan; Faculty of Life and Environmental Sciences, University of Tsukuba Tsukuba, Ibaraki 305-8572, Japan; Research Center for Bioscience and Nanoscience (CeBN), Japan Agency for Marine-Earth Science and Technology (JAMSTEC), Yokosuka, Kanagawa 237-0061, Japan; Graduate School of Agricultural and Life Sciences, The University of Tokyo, Bunkyo, Tokyo 113-8657, Japan; Collaborative Research Institute for Innovative Microbiology, The University of Tokyo, Bunkyo, Tokyo 113-8657, Japan; Institute for Extra-cutting-edge Science and Technology Avant-garde Research (X-star) Japan Agency for Marine-Earth Science and Technology (JAMSTEC), Yokosuka, Kanagawa 237-0061, Japan; Center for Sustainable Resource Science, RIKEN, Wako, Saitama 351-0198, Japan; Graduate School of Science and Technology University Tsukuba, Tsukuba, Ibaraki 305-8572, Japan; Institute for Extra-cutting-edge Science and Technology Avant-garde Research (X-star) Japan Agency for Marine-Earth Science and Technology (JAMSTEC), Yokosuka, Kanagawa 237-0061, Japan; Graduate School of Agriculture, Kyoto University, Kyoto, Kyoto 606-8502, Japan; Institute of Low Temperature Science, Hokkaido University, Sapporo, Hokkaido 060-0819, Japan; Research Center for Bioscience and Nanoscience (CeBN), Japan Agency for Marine-Earth Science and Technology (JAMSTEC), Yokosuka, Kanagawa 237-0061, Japan

**Keywords:** autotroph, carbon fixation, thermophile, hydrothermal environment, stable isotope probing, WL pathway, rTCA cycle

## Abstract

The bacterial-type Wood–Ljungdahl (WL) pathway and reductive tricarboxylic acid (rTCA) cycle are the dominant chemolithotrophic CO_2_ fixation pathways in bacteria inhabiting aphotic geothermal and deep-sea hydrothermal ecosystems. However, the activity of these bacterial metabolic systems in ecosystems with available organic carbons remains unclear. Here, we examined the impact of extracellular acetate on the CO_2_-fixation pathways of three thermophilic hydrogen-oxidizing and non-acetogenic bacteria using ^13^C tracer-based metabolomics. Under chemolithoautotrophic conditions, *Thermodesulfatator indicus* and *Hydrogenobacter thermophilus* fixed CO_2_ through the WL pathway and rTCA cycle, respectively, whereas *Thermovibrio ammonificans*, which has been suggested to operate both of these pathways, exhibited significant CO_2_ fixation through only the rTCA cycle. Under chemolithomixotrophic conditions with acetate, *H. thermophilus* and *T. ammonificans* assimilated both CO_2_ and acetate via the rTCA cycle. In contrast, acetate suppressed CO_2_ fixation through the WL pathway in *T. indicus* and was used as the primary carbon source under chemolithomixotrophic conditions. These results suggest that the contribution of the WL pathway for CO_2_ fixation might be overestimated in ecosystems where acetate is available. Moreover, the present findings indicate that simultaneous CO_2_ fixation through both the WL pathway and rTCA cycle in a cell, which has been proposed as a possible metabolic strategy for CO_2_-fixation in ancestral life, is not advantageous in extant microorganisms.

## Graphical Abstract

**Graphical Abstract:**
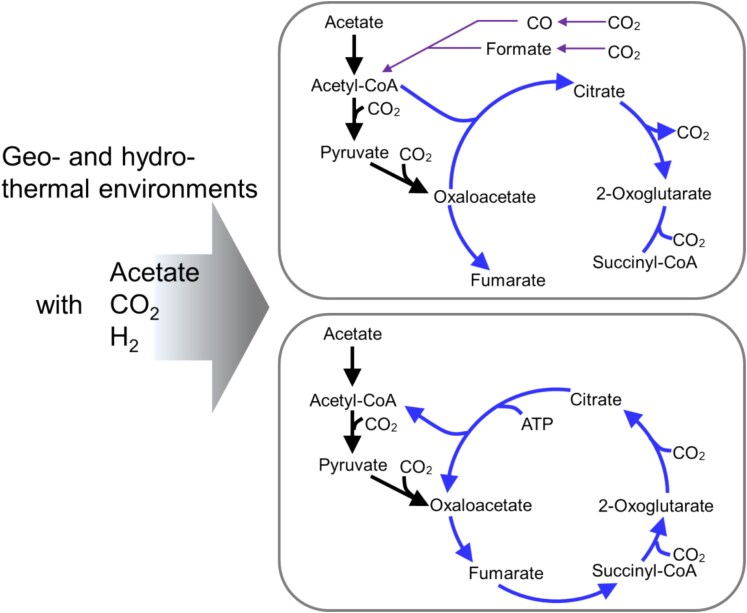

## Introduction

Chemolithoautotrophic primary production is essential to sustain aphotic geothermal and deep-sea hydrothermal ecosystems [1], which are recognized as modern analogs of early ecosystems on Earth [2–4]. The Wood–Ljungdahl (WL) pathway and reductive tricarboxylic acid (rTCA) cycle, the former of which is also called the reductive acetyl-CoA pathway, are the dominant bacterial CO_2_ fixation pathways in these geothermal and hydrothermal environments [1]. However, as the WL pathway and rTCA cycle are fundamentally reversible, it remains unclear if these pathways are capable of CO_2_ fixation in ecosystems where extracellular organic carbons are readily available [5].

Most extant bacterial WL pathways are found in acetogens that utilize CO_2_ as both an electron acceptor and carbon source [6, 7], and direct observation of this pathway in non-acetogens is limited. One exception is *Desulfotomaculum acetoxidans*, a mesophilic bacterium isolated from piggery waste that has a functional WL pathway which operates both reductively and oxidatively using hydrogen and acetate, respectively, as electron donors [8–10]. Putative genes for the bacterial WL pathway enzymes have also been identified in hydrogenotrophic sulfate reducers, including members of the class *Thermodesulfobacteria* [11, 12], suggesting that thermophilic, chemolithoautotrophic non-acetogens play a significant role as primary producers through operation of the WL pathway for CO_2_ fixation in geothermal and hydrothermal environments [11, 12]. However, the bacterial WL pathway function has not been demonstrated in obligately hydrogenotrophic and thermophilic non-acetogenic bacteria under chemolithoautotrophic or chemolithomixotrophic conditions.

The rTCA cycle is a variant of the TCA cycle, which is also called the citric acid cycle or Krebs cycle [13]. Conventionally, the direction of TCA cycle reactions is predicted by key enzymes in this pathway, particularly those catalyzing the cleavage of citrate (Table S1) [14, 15]. Citrate cleavage in the rTCA cycle is generally thought to be catalyzed by ATP-citrate lyase (ACL) [16, 17] or by citryl-CoA synthestase (CCS) and citryl-CoA lyase (CCL) in a two-step reaction [18, 19]. Although these enzymes have been recognized as signatures of the rTCA cycle, multiple lines of evidence has revealed that the TCA cycle direction is controlled not only by the key enzyme(s), but also by various external and internal factors, including available carbon sources, partial pressure of CO_2_, and amount and/or kinetic parameters of enzymes involved in the cycle [15, 20–23]. For instance, *Thermosulfidibacter takaii* [22, 24], *Desulfurella acetivorans* [15, 23], and other thermophilic and hydrogen-oxidizing bacteria [23] isolated from hydrothermal or geothermal systems operate the TCA cycle in the reductive direction using ATP-independent citrate synthase (CS) at chemolithoautotrophic growth conditions with high-partial pressures of CO_2_. Notably, the TCA cycle operates partially or completely oxidatively when *T. takaii* is grown chemolithomixotrophically with organic acids as carbon sources even under 20% CO_2_ (v/v) of headspace gas [22]. As another example, the mesophilic sulfate reducer *Desulfobacter postgatei* operates the TCA cycle oxidatively with ACL to generate ATP via the oxidation of acetate [17].

The WL pathway and rTCA cycle are considered to be early-evolved CO_2_ fixation systems among the seven known CO_2_ fixation pathways [25–31]. In addition, the occurrence of both the WL pathway and rTCA cycle in a cell is proposed as a possible ancestral form of CO_2_ fixation system based on comparative genomic analyses [28, 32, 33]. In fact, chemolithoautotrophs that fix CO_2_ through the WL pathway likely also operate at least part of the TCA cycle to obtain the essential cellular metabolic building blocks [28] other than acetyl-CoA, including pyruvate, oxaloacetate (OAA), succinyl-CoA (or succinate), and 2-oxoglutarate (2-OG). However, cooperation of the WL pathway and complete TCA cycle in either working direction has not been demonstrated in any cellular system to date. The possibility that a thermophilic hydrogen-oxidizer *Thermovibrio ammonificans* [34] operates the WL pathway as well as the rTCA cycle has been proposed based on the combined genomic and proteomic analyses nonetheless this bacterium lacks the candidate genes for 10-formyl-tetrahydrofolate synthetase and acetyl-CoA synthase [32]. Although the former one could be replaced by alternative enzymes genes [32], the lack of candidate genes for acetyl-CoA synthase, a key enzyme in the WL pathway, has remained unsolved. Furthermore, *in silico* kinetic simulations suggest that increased acetyl-CoA influx caused by a functioning WL pathway would negatively impact the reductive operation of the TCA cycle with CS [35]; however, the ability of the WL pathway and rTCA cycle to operate with ACL has not been theoretically examined.

Members of class *Thermodesulfobacteria* in the phylum *Thermodesulfobacteriota* and classes *Aquificia* and *Desulfurobacteriia* in the phylum *Aquificota* are hydrogenotrophic, extremely thermophilic (optimal growth temperature exceeds 70°C), and comprise the dominant bacterial populations in geothermal and hydrothermal ecosystems. *Thermodesulfobacteria* bacteria are predicted to harbor the WL pathway based on gene homology [11], and *Aquificota* bacteria have a functional rTCA cycle for inorganic carbon fixation [36]. Moreover, *T. ammonificans* in *Desulfurobacteriia* is thought to operate both the WL pathway and rTCA cycle, as described above [32]. Here, we examined the activity of these CO_2_ fixation pathways in *Thermodesulfatator indicus* in *Thermodesulfobacteriota* [12], and *T. ammonificans* and *Hydrogenobacter thermophilus* in *Aquificia* [37–39], under chemolithoautotrophic and chemilithomixotrophic conditions using ^13^C tracer-based metabolomics [22] to evaluate their operation in geothermal and hydrothermal ecosystems and to gain insight into the origin and evolution of central carbon metabolism.

## Materials and methods

### Strains and growth conditions

*Thermodesulfatator indicus* JCM 11887^T^ (=CIR29812), *T. ammonificans* JCM 12110 ^T^ (=HB-1), and *H. thermophilus* JCM 7687^T^ (=TK-6) were provided by RIKEN BRC through the National BioResource Project of the MEXT, Japan. Glass test tubes with volumes of 17 and 20 ml (φ16 × 125 and φ16 × 150 mm, IWAKI, Japan) were used for the cultivation of *T. ammonificans* and *T. indicus*, respectively, and a 100-ml glass serum bottle (φ40.5 × 128.0 mm, Maruemu, Japan) was used to cultivate *H. thermophilus*.

*Thermodesulfatator indicus*, *T. ammonificans*, and *H. thermophilus* were cultivated in inorganic medium (3, 4, and 5 ml, respectively) with H_2_ and CO_2_ as energy and carbon sources, respectively at 70°C. See the Supporting Information for details.

### Isotopologue and isotopomer analyses

All ^13^C-labeled regents (purity of >99%) used in this study were purchased from Cambridge Isotope Laboratories (Tewksbury, MA, USA).

See the Supporting Information for details on the cell cultivation with ^13^C compounds. To examine CO_2_ incorporation in *T. indicus* cells grown chemolithoautotrophically, cells were harvested 7 h after the addition of 10% (v/v of gas phase) ^13^CO_2_ during the late exponential phase. To examine the incorporation of CO_2_ in *T. indicus* cells grown chemolithomixotrophically with acetate, ^12^C_2_ sodium acetate (2 mM final concentration) and 2 mL of ^13^CO_2_, which corresponded to 5% v/v of the final gas phase (H_2_:CO_2_) and 25% of all CO_2_ in the test tube, was added prior to the cultivation. To examine the incorporation of acetate and formate in *T. indicus* cells, [1,2–^13^C_2_] sodium acetate or ^13^C sodium formate (2 mM final concentration), respectively, was added to the inorganic medium prior to cultivation. To examine CO incorporation in *T. indicus* cells, 1 ml of ^13^CO was added to the gas phase to give a final CO:CO_2_ ratio of 1:3.

To examine CO_2_ incorporation in *T. ammonificans* cells grown chemolithoautotrophically, cells in the late exponentially phase were harvested 3 h after the addition of 10% ^13^CO_2_ (v/v of the final gas phase). To examine ^13^CO incorporation in *T. ammonificans* cells, 2.4 ml of ^13^CO was added to the H_2_ atmosphere and a gas mixture of 80% H_2_ and 20% CO_2_ was added to 0.20 MPa. After a 16-h incubation, the cells were harvested by centrifugation. To examine acetate incorporation in *T. ammonificans* cells grown chemolithomixotrophically, [1,2–^13^C_2_] sodium acetate (10 mM final concentration) was added to the inorganic medium prior to cultivation.

To obtain labeled amino acids from *H. thermophilus* cells grown chemolithoautotrophically or chemolithomixotrophically, 10 mL of ^13^CO_2_, which corresponded to ~10% (v/v) of the gas phase, was added to exponential phase cultures ~6 h after inoculation. Cells were harvested by centrifugation 2.5 h after the addition of ^13^CO_2_. To examine the incorporation of acetate in *H. thermophilus* cells, [1,2–^13^C_2_] sodium acetate (10 mM final concentration) was added to the inorganic medium prior to cultivation, and cells were harvested by centrifugation in the late exponential phase.

The harvested cells were stored at −80°C until needed for further processing. To prepare protein-derived amino acids, the cells were hydrolyzed in 12 N HCl at 110°C and examined using a ZipChip capillary electrophoresis (CE) system (908 Devices, Boston, MA, USA) coupled with an Orbitrap Fusion Tribrid mass spectrometer (Thermo Fisher Scientific, Waltham, MA) as described previously [40]. The obtained MS data was analyzed using Qual Browser in Xcalibur (version 4.3.73.11). The relative abundance of isotopologues and isotopomers of each amino acid was determined from CE-MS- and CE-MS/MS-based data, respectively.

## Results

### Predicted metabolic pathways of the strains used in this study

**Figure 1:**
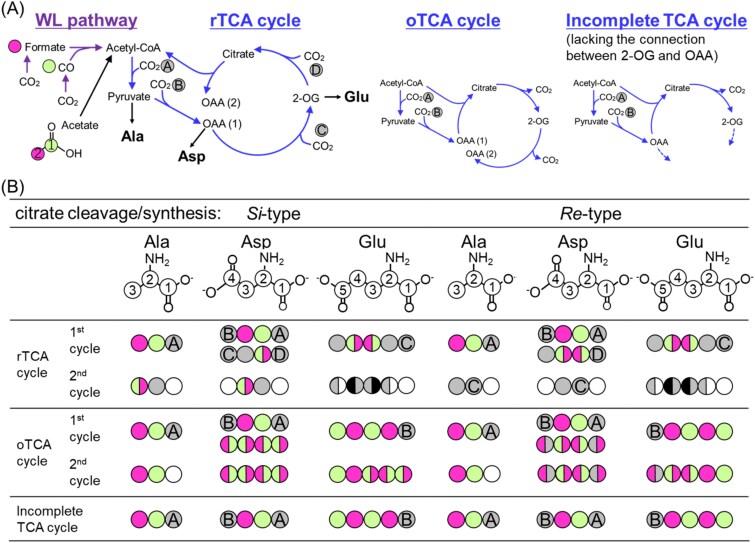
Schematics of the WL pathway and reductive, oxidative and incomplete TCA cycles (A), and the expected labeling patterns of metabolites (B). (A) Reactions included in the WL pathway and TCA cycle are shown with purple and blue arrows, respectively. rTCA cycle: reductive TCA cycle; oTCA cycle: oxidative TCA cycle; OAA: oxaloacetate; 2-OG: 2-oxoglutarate. (B) Carbons derived from CO, formate, and acetate are shown with light green, magenta and light green (C1 of acetate) and magenta (C2 of acetate), respectively. Carbons fixed through the first cycle of the TCA cycle are highlighted in gray and labeled from “A” to “D” based on the reactions shown in (A). Black circles indicate carbons derived from CO or formate. Note that the labeling pattern differs based on the stereochemistry of the citrate cleavage/synthesis enzymes. Upper and lower Asps are derived from OAA (1) and OAA (2), respectively, in (A).

The distribution of genes encoding WL pathway and TCA cycle enzymes varied among the genomes of the three tested species. *Thermodesulfatator indicus* encoded a complete set of WL pathway genes, whereas the TCA cycle gene set appeared to lack genes for the enzymatic interconversion between 2-OG and OAA (Fig. 1A) (see also the Supplementary Text for details, Fig. S1 and Tables S2 and S3). *Thermovibrio ammonificans* possessed all of the genes for the rTCA cycle, and also harbored most of the candidate genes for WL pathway enzymes, with the exception of acetyl-CoA synthase (Figs 1A and S1) [32]. *Hydrogenobacter thermophilus*, which serves as a model organism for understanding the machinery of the rTCA cycle [41–45], lacked most of the genes related to the WL pathway (Figs 1A and S1) [32].

Among the three examined strains, variations in the gene involved in citrate synthesis/cleavage reactions were also identified. CS is classified into *Si*- and *Re*-types based on the stereospecificity of the binding manner of acetyl-CoA and OAA (Fig. S2), and the reactions catalyzed by these enzymes can be determined by isotopomer structure using a tracer-based metabolomics approach (Fig. 1B) [46, 47]. *Thermodesulfatator indicus* has a homolog of *Re*-CS (Fig. S3; see also details in the Supplementary Text), whereas *T. ammonificans* and *H. thermophilus* harbor ACL and CCS/CCL, respectively, and lack CS (Table S2). CCL is the ancestral form of ACL and *Si*-CS [48, 49] and predicted to have the same stereochemistry as *Si*-CS [23, 45]. All the three strains have candidate gene for acetyl-CoA synthetase, which catalyzes the conversion of acetate to acetyl-CoA (Fig. S1).

### Effects of acetate on growth and protein expression by the three hydrogen oxidizers

*Thermodesulfatator indicus, T. ammonificans*, and *H. thermophilus* were cultivated chemolithoautotrophically with H_2_ and CO_2_ as energy and carbon sources, respectively, and also chemolithomixotrophically using acetate as a carbon source in addition to CO_2_ with hydrogen as an energy source. Under chemolithomixotrophic conditions, the growth of *T. indicus* and *H. thermophilus* was slightly stimulated and the final cell density was increased by 20% compared to that obtained under chemolithoautotrophic conditions (Fig. S4), as reported previously [12, 50]. In contrast, the addition of acetate had no discernible effect on the growth rate or final cell density of *T. ammonificans* (Fig. S4).

Gene expression related to the WL pathway and TCA cycle was also examined by shotgun proteomics. In *T. indicus*, all of the enzymes in the WL pathway and an incomplete set of TCA cycle enzymes were expressed under chemolithoautotrophic and chemolithomixotrophic conditions (Table S4). In *H. thermophilus*, all TCA cycle enzymes, except for a few subunits of heteromeric proteins, were detected under both chemolithoautotrophic and chemolithomixotrophic conditions (Table S5). The expression of both WL pathway and TCA cycle proteins in *T. ammonificans* under chemolithoautotrophic condition has been reported previously [32].

### Carbon flow in *T. indicus*

The operation and working direction of the reactions of the WL pathway and/or TCA cycle were examined in all three species using tracer-based metabolomics. Tables 1 and 2 summarize the pattern of isotopomers and isotopologues of the amino acids, Ala, Asp and Glu, which were obtained by protein hydrolysis in cells grown with ^13^C-labeled substrates. The patterns of Ala, Asp and Glu reflected those of their precursors: pyruvate, OAA, and 2-OG, respectively [40].

**Table 1:** The mass isotopomer distributions of alanine, aspartate, and glutamate from CE-MS analysis.

		Natural abundance of isotopes		*T. indicus*							*H. thermophilus*					*T. ammonificans*			
				^13^CO_2_ + CO_2_	^13^CO + CO_2_	[^13^C]formate + CO_2_	[1,2-^13^C_2_]acetate + CO_2_	acetate + ^13^CO_2_ + CO_2_	CO_2_ (negative control)		^13^CO_2_ + CO_2_	[1,2-^13^C_2_]acetate + CO_2_	acetate +^13^CO_2_ + CO_2_	CO_2_ (negative control)		^13^CO_2_ + CO_2_	^13^CO + CO_2_	[1,2-^13^C_2_]acetate + CO_2_	CO_2_ (negative control)
Amino acid		abundance (%)		abundance (%)	abundance (%)	abundance (%)	abundance (%)	abundance (%)	abundance (%)		abundance (%)	abundance (%)	abundance (%)	abundance (%)		abundance (%)	abundance (%)	abundance (%)	abundance (%)
Ala	^13^C_0_	96.7		40.0	91.9	75.9	34.7	66.6	97.1		73.6	39.8	80.6	96.9		62.3	97.7	83.9	97.7
	^13^C_1_	3.2		26.0	8.1	23.4	3.6	30.7	2.9		13.5	15.1	17.5	3.1		29.1	2.3	5.8	2.3
	^13^C_2_	0.0		26.3	N.D.	0.8	61.3	2.7	N.D.		10.2	44.9	1.8	N.D.		8.4	N.D.	10.4	N.D.
	^13^C_3_	0.0		7.7	N.D.	N.D.	0.4	N.D.	N.D.		2.8	0.2	N.D.	N.D.		0.2	N.D.	N.D.	N.D.
Asp	^13^C_0_	95.7		35.3	90.6	78.2	45.7	49.5	96.6		67.9	49.6	68.1	96.0		53.8	94.9	90.1	96.7
	^13^C_1_	4.3		18.7	9.4	21.8	4.1	39.4	3.4		12.0	24.6	22.2	4.0		32.1	5.1	6.9	3.3
	^13^C_2_	0.1		26.3	N.D.	N.D.	49.9	10.3	N.D.		12.2	25.8	8.7	N.D.		12.8	N.D.	3.0	N.D.
	^13^C_3_	0.0		15.9	N.D.	N.D.	0.3	0.8	N.D.		6.4	N.D.	0.9	N.D.		1.3	N.D.	N.D.	N.D.
	^13^C_4_	0.0		3.8	N.D.	N.D.	N.D.	N.D.	N.D.		1.5	N.D.	0.0	N.D.		N.D.	N.D.	N.D.	N.D.
Glu	^13^C_0_	94.6		31.3	86.4	64.8	25.0	61.4	94.8		62.9	48.6	60.1	94.7		42.8	93.7	87.3	96.1
	^13^C_1_	5.3		13.6	13.6	22.9	3.5	32.2	5.2		10.5	25.0	22.1	5.3		34.0	6.3	9.2	3.9
	^13^C_2_	0.1		23.0	N.D.	11.4	28.8	5.6	N.D.		11.8	25.4	13.1	N.D.		17.7	N.D.	3.4	N.D.
	^13^C_3_	0.0		20.2	N.D.	0.9	5.0	0.8	N.D.		9.8	1.0	4.1	N.D.		4.9	N.D.	0.1	N.D.
	^13^C_4_	0.0		9.9	N.D.	N.D.	37.3	N.D.	N.D.		4.2	N.D.	0.6	N.D.		0.6	N.D.	N.D.	N.D.
	^13^C_5_	0.0		1.9	N.D.	N.D.	0.4	N.D.	N.D.		0.8	N.D.	0.1	N.D.		0.0	N.D.	N.D.	N.D.

N.D., not detected.

**Table 2:** The isotopomer pattern of alanine, aspartate, and glutamate from CE-MS/MS analysis.

		Isotopomer pattern		Natural abundance of isotopes		*T. indicus*							*H. thermophilus*					*T. ammonificans*			
		No. of ^13^C				^13^CO_2_ + CO_2_	^13^CO + CO_2_	[^13^C]formate + CO_2_	[1,2-^13^C_2_]acetate + CO_2_	acetate +^13^CO_2_ + CO_2_	CO_2_ (negative control)		^13^CO_2_ + CO_2_	[1,2-^13^C_2_]acetate + CO_2_	acetate +^13^CO_2_ + CO_2_	CO_2_ (negative control)		^13^CO_2_ + CO_2_	^13^CO + CO_2_	[1,2–^13^C_2_]acetate + CO_2_	CO_2_ (negative control)
Amino acid		C-1	Others	abundance (%)		abundance (%)	abundance (%)	abundance (%)	abundance (%)	abundance (%)	abundance (%)		abundance (%)	abundance (%)	abundance (%)	abundance (%)		abundance (%)	abundance (%)	abundance (%)	abundance (%)
Ala	^13^C_0_	0	0	96.7		40.0	91.9	75.9	34.7	66.6	97.1		73.6	39.8	80.6	96.9		62.3	97.7	83.9	97.7
	^13^C_1_	1	0	1.1		8.4	1.1	2.2	0.3	24.5	1.0		4.5	0.4	13.1	1.0		9.4	0.8	0.9	0.8
		0	1	2.2		17.6	7.0	21.2	3.3	6.2	2.0		8.9	14.7	4.5	2.1		19.7	1.5	4.9	1.5
	^13^C_2_	1	1	0.0		16.9	N.D.	0.4	0.1	2.0	N.D.		6.7	0.2	1.5	N.D.		5.5	N.D.	0.1	N.D.
		0	2	0.0		9.4	N.D.	0.4	61.3	0.7	N.D.		3.5	44.7	0.4	N.D.		3.0	N.D.	10.3	N.D.
	^13^C_3_	1	2	0.0		7.7	N.D.	N.D.	0.4	N.D.	N.D.		2.8	0.2	N.D.	N.D.		0.2	N.D.	N.D.	N.D.
Asp	^13^C_0_	0	0	95.7		35.3	90.6	78.2	45.7	49.5	96.6		67.9	49.6	68.1	96.0		53.8	94.9	90.1	96.7
	^13^C_1_	1	0	1.1		4.6	1.3	2.5	0.4	16.4	0.9		3.4	0.9	7.4	1.0		7.6	1.2	1.2	1.1
		0	1	3.2		14.1	8.1	19.2	3.6	23.0	2.5		8.6	23.7	14.9	3.0		24.5	3.9	5.8	2.2
	^13^C_2_	1	1	0.0		13.4	N.D.	N.D.	0.0	8.6	N.D.		5.6	N.D.	6.6	N.D.		6.5	N.D.	N.D.	N.D.
		0	2	0.0		13.0	N.D.	N.D.	49.9	1.8	N.D.		6.6	25.8	2.1	N.D.		6.3	N.D.	3.0	N.D.
	^13^C_3_	1	2	0.0		12.3	N.D.	N.D.	0.3	0.8	N.D.		5.0	N.D.	0.9	N.D.		1.3	N.D.	N.D.	N.D.
		0	3	0.0		3.6	N.D.	N.D.	N.D.	N.D.	N.D.		1.4	N.D.	N.D.	N.D.		N.D.	N.D.	N.D.	N.D.
	^13^C_4_	1	3	0.0		3.8	N.D.	N.D.	N.D.	N.D.	N.D.		1.5	N.D.	0.0	N.D.		N.D.	N.D.	N.D.	N.D.
Glu	^13^C_0_	0	0	94.6		31.3	86.4	64.8	25.0	61.4	94.8		62.9	48.6	60.1	94.7		42.8	93.7	87.3	96.1
	^13^C_1_	1	0	1.1		2.3	5.1	2.1	N.D.	2.3	0.7		3.1	1.1	8.1	1.4		6.2	1.7	1.0	0.6
		0	1	4.2		11.4	8.6	20.8	3.5	30.0	4.5		7.4	23.9	14.0	4.0		27.8	4.6	8.2	3.2
	^13^C_2_	1	1	0.0		8.4	N.D.	0.8	13.2	2.2	N.D.		4.1	0.5	8.1	N.D.		7.1	N.D.	N.D.	N.D.
		0	2	0.1		14.6	N.D.	10.6	15.5	3.5	N.D.		7.8	24.9	4.9	N.D.		10.6	N.D.	3.4	N.D.
	^13^C_3_	1	2	0.0		12.1	N.D.	0.4	3.4	0.8	N.D.		5.8	0.4	3.3	N.D.		3.6	N.D.	N.D.	N.D.
		0	3	0.0		8.2	N.D.	0.5	1.7	N.D.	N.D.		4.0	0.6	0.8	N.D.		1.3	N.D.	N.D.	N.D.
	^13^C_4_	1	3	0.0		7.7	N.D.	N.D.	37.3	N.D.	N.D.		3.5	N.D.	0.5	N.D.		N.D.	N.D.	N.D.	N.D.
		0	4	0.0		2.2	N.D.	N.D.	N.D.	N.D.	N.D.		0.7	N.D.	0.1	N.D.		N.D.	N.D.	N.D.	N.D.
	^13^C_5_	1	4	0.0		1.9	N.D.	N.D.	0.4	N.D.	N.D.		0.8	N.D.	0.1	N.D.		0.0	N.D.	N.D.	N.D.

N.D., not detected.

*T. indicus* cells grown chemolithoautotrophically with ^13^CO_2_ incorporated single or multiple ^13^C molecules in both the C-1 and non-C-1 positions of Ala and Asp (Table 2). In contrast, when ^13^CO or [^13^C] formate was added together with ^12^CO_2_ in the culture medium, incorporation of single ^13^C molecules into non-C-1 positions was observed in most labeled Ala and Asp (Table 2). These results were consistent with the expected labeling patterns, which are based on the assumption that CO_2_, CO, and formate are directly incorporated into acetyl-CoA through the WL pathway, and that acetyl-CoA is carboxylated to pyruvate and then further carboxylated to form OAA (Fig. 1A, Fig. S1). The oxidation of CO to CO_2_ was negligible, whereas ~5% of the formate incorporated into the cells was oxidized to CO_2_ (complete experimental details and results are provided in the Supplementary Text and Fig. S5). Taken together, these results demonstrate that the WL pathway functions as a chemolithoautotrophic CO_2_ fixation pathway in the extremely thermophilic and non-acetogenic bacterium *T. indicus*.

The working direction of the reactions of the TCA cycle in *T. indicus* was next assessed based on the labeling pattern of Glu and the following rationale. If 2-OG is synthesized from OAA through the rTCA cycle, no ^13^C would be observed in the C-1 position of Glu when *T. indicus* is cultivated with ^13^CO or [^13^C] formate in cases when the CO and formate are not oxidized to CO_2_ (Fig. 1B, rTCA cycle with *Re*-type citrate cleavage/synthesis; Fig. S6B). In contrast, if 2-OG is synthesized through the oxidative TCA (oTCA) cycle, the C-1 position of Glu would be labeled with ^13^C derived from ^13^CO, but not from [^13^C] formate (Fig. 1B, Fig. S7B). We found that the ratio of ^13^C_1_ Glu with ^13^C in the C-1 position was 5.1% and 2.1% in *T. indicus* cells cultured in the presence of ^13^CO and [^13^C] formate, respectively (Table 2). The low percentage of ^13^C_1_ Glu with ^13^C in the C-1 position in cultures with [^13^C] formate was likely due to the incorporation of CO_2_ derived from oxidized [^13^C] formate. These findings suggest that 2-OG was synthesized through the oTCA cycle. Notably, however, the connection and working direction among OAA, succinyl-CoA and 2-OG could not be determined based on the isotopomer patterns obtained in this study.

The impact of acetate on the WL pathway was also analyzed under chemolithomixotrophic conditions. If all cellular acetyl-CoA is synthesized from CO_2_ through the WL pathway, it is expected that in the presence of ^13^CO_2_ and ^12^CO_2_, the ratio of ^13^C_1_ Ala, which is derived from pyruvate through acetyl-CoA, with ^13^C at the C-1 position to the C-2 or C-3 position would be 1:2. In *T. indicus* cells grown chemolithoautotrophically under these conditions, the observed ratio was nearly 1:2 (8.4:17.6). In contrast, in cultures supplemented with non-labeled acetate and ^13^CO_2_ (^13^CO_2_: ^12^CO_2_ = 1:3), the ratio was altered to approximately 4:1 (24.5:6.2; Table 2), indicating that more than 85% of acetyl-CoA was derived from the external acetate source. Considering that the cell yield under chemolithomixotrophic conditions with acetate increased by only 20% compared to the yield resulting from chemolithoautotrophic growth, CO_2_ fixation through the WL pathway was likely suppressed by the increased acetyl-CoA influx from acetate.

The working direction of the reactions of the TCA cycle in *T. indicus* cells grown chemolithomixotrophically with [1,2–^13^C_2_] acetate and ^12^CO_2_ was next investigated. Under these conditions, 37.3% of Glu was in the form of ^13^C_4_, with the C-1 carboxyl group being labeled with ^13^C (Table 2). The observed labeling pattern confirmed that 2-OG was synthesized with acetyl-CoA and OAA through oxidative operation of the TCA cycle (Fig. 1B, see the Supporting Information for details). The labelling patterns of Asp (49.9% ^13^C_2_ and 0.3% ^13^C_3_) indicated that the majority of OAA was synthesized through the direct carboxylation of pyruvate, but not from ^13^C_4_ 2-OG via the oTCA cycle. Collectively, these results reveal that the TCA cycle is incomplete and bifurcated in *T. indicus*, and that enzymatic reactions between 2-OG and OAA do not occur under autotrophic or mixotrophic conditions, as would be expected based on the genome sequence (Fig. 1A, Fig. S1 and Fig. S8).

### Carbon flow in *T. ammonificans*

**Figure 2:**
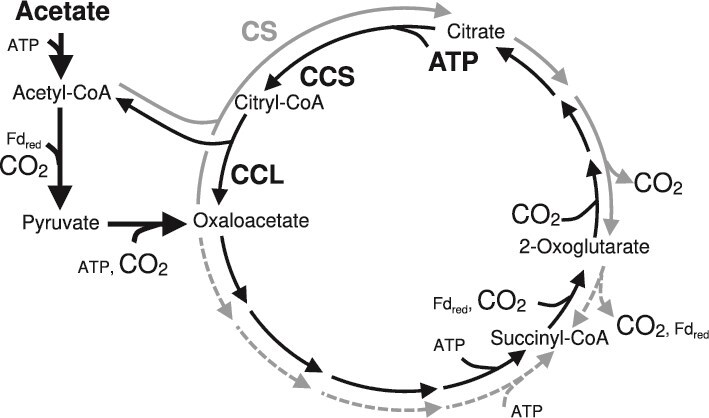
Flow of the TCA cycle under chemolithomixotrophic conditions with acetate in *H. thermophilus* and *T. takaii*. *H. thermophilus* (thin black arrows) and *T. takaii* (gray arrows) have ATP-dependent (CCS/CCL) and ATP-independent (CS) citrate cleavage/synthesis systems, respectively. Common reactions in the two organisms are indicated by thick black arrows. Dotted arrows indicate the expected working direction of the TCA cycle, which cannot be theoretically determined based on isotopomer patterns. CCS: Citryl-CoA synthetase; CCL: Citryl-CoA lyase; CS: Citrate synthase; Fd_red_: reduced ferredoxin.

When *T. ammonificans* was cultivated with ^13^CO and non-labeled CO_2_, no significant incorporation of ^13^C in Ala, Asp, and Glu was observed, in contrast to the case of *T. indicus* (Fig. 2, Table 1), indicating that the WL pathway had no contribution to CO_2_ fixation. Under chemolithoautotrophic conditions, the labeling patterns of *T. ammonificans* cells grown with ^13^CO_2_ and ^12^CO_2_ (1:2) were consistent with those expected by operation of the rTCA cycle with ACL. Namely, the observed abundance of ^13^C_2_ and ^13^C_3_ Glu (17.7% and 4.9%, respectively) (Table 2) suggests that 2-OG was only synthesized through reductive operation of the TCA cycle, assuming that the proportion of acetyl-CoA derived from the WL pathway was negligible or absent (Fig. 1, Figs S6A and S7A). The formation of ^13^C_2_ Ala, which had ^13^C in both C-1 and C-2 or C-3 positions (5.5%), and ^13^C_2_ Asp, which had two ^13^C in positions other than the C-1 carboxyl group (6.3%), can be explained by a second turn of the rTCA cycle. Moreover, a lower abundance of ^13^C_2_ Ala with ^13^C in both the C-2 and C-3 positions was detected (3.0%), suggesting that this compound may be a signature of the third turn of the rTCA cycle.

We also examined the assimilation of [1,2–^13^C_2_] acetate by *T. ammonificans* in the presence of H_2_ and ^12^CO_2_. ^13^C_2_ Ala comprised 10.4% of total Ala, indicating that acetate was incorporated into the cells (Table 1). Approximately 3% of ^13^C excess was observed in ^13^C_1_ Asp and Glu, indicating that the rTCA cycle was functional (Table 1). As acetate had no impact on the growth of *T. ammonificans*, which also exhibited lower acetate assimilation compared to *T. indicus* and *H. thermophilus*, we concluded that *T. ammonificans* passively assimilated acetate and that the rTCA cycle was not significantly affected by the acetyl-CoA influx from the external acetate source. The passive assimilation of acetate by in *T. ammonificans* is consistent with the lack of a candidate gene for a cation/acetate symporter (KEGG ORTHOLOGY: K14393), which was identified in *T. indicus* and *H. thermophilus* (Thein_1042 and HTH_1561, respectively).

### Carbon flow in *H. thermophilus*

The labeling patterns of *H. thermophilus* cells grown with ^13^CO_2_ and ^12^CO_2_ were consistent with those produced by a functional rTCA cycle that includes CCS and CCL, which has the same stereochemistry with that of *Si*-CS (Fig. S6A). The detection of ^13^C_3_ and ^13^C_4_ Glu and ^13^C_2_ Asp with two ^13^C in carboxyl groups located at positions other than C-1 (6.6%) confirmed operation of the rTCA cycle, but not the oTCA cycle (Fig. 1 and Table 1). Further, the production of ^13^C_2_ Ala with ^13^C in both the C-1 and C-2 or C-3 positions (6.7%) would be expected in the second turn of the rTCA cycle (Table 2). The ^13^C labeling patterns with ^13^CO_2_ confirmed that *H. thermophilus* operates the rTCA cycle under the tested chemolithoautotrophic conditions.

The working direction of the TCA cycle in *H. thermophilus* was also examined under chemolithomixotrophic conditions with supplemental acetate. In cells grown with [1,2-^13^C_2_] acetate and ^12^CO_2_, more than 50% of Ala, Asp, and Glu contained one or two ^13^C atoms, confirming that *H. thermophilus* actively incorporated acetate into cellular compounds. The production of ^13^C_1_ Ala, Asp, and Glu would occur during the second cycle of the rTCA cycle, but would not be formed by the oTCA cycle or an incomplete TCA cycle (Fig. 1). Notably, the amount of ^13^C_4_ Glu, which can be produced only through the oTCA cycle, was negligible despite the high ratio of [1,2-^13^C_2_] acetate incorporation by *H. thermophilus*. The operation of the rTCA cycle was also supported by isotopologue analysis of cells grown with non-labeled acetate and ^13^CO_2_. Under these conditions, the detection of Ala with ^13^C in C-1, Asp with ^13^C in non-C-1, and Glu with ^13^C in C-1 positions indicated the carboxylation of acetyl-CoA, pyruvate, and succinyl-CoA, respectively.

## Discussion

### Impact of acetate on tricarboxylic acid cycle variants

Under chemolithomixotrophic conditions, in which H_2_, acetate and high partial pressures (20%) of CO_2_ are available, the impact of acetate on the TCA cycle, particularly on citrate cleavage/synthesis reactions, varies depending on the types of enzymes catalyzing these reactions. The findings from the present study revealed that the hydrogen-oxidizing bacteria *H. thermophilus* (Fig. 2) and *T. ammonificans* operate the rTCA cycle, including the associated citrate cleavage reaction(s), as was reported for the green sulfur bacterium *Chlorobaculum tepidum* [51]. Thus, the reductive operation of the TCA cycle with ATP-dependent citrate cleavage reactions in cells that utilize acetate and CO_2_ as carbon sources appears to be a shared strategy among chemolithotrophs and photolithotrophs.

In contrast to the above hydrogen-oxidizing bacteria, citrate synthesis with CS occurs in the obligate hydrogenotroph *T. takaii* (Fig. 2) and facultative hydrogenotroph *D. acetivorans* under chemolithomixotrophic conditions in the presence acetate, whereas their TCA cycle proceeds in the reductive direction with citrate cleavage with CS under chemolithoautotrophic conditions [15, 22]. The marked difference in the operation of the TCA cycle under chemolithomixotrophic conditions with acetate can be explained by the thermodynamic properties of the enzymes used for citrate cleavage/synthesis. ATP-dependent citrate cleavage by ACL (*T. ammonificans* and *C. tepidum*) or CCS/CCL (*H. thermophilus*) is thermodynamically close to neutral (ΔG^0’^ = 5.6 kJ/mol), whereas ATP-independent citrate cleavage with CS (*T. takaii* and *D. acetivorans*) is highly endergonic (ΔG^0’^ = 37.4 kJ/mol) [17] (Table S1). Thus, citrate cleavage is more likely to occur in *H. thermophilus, T. ammonificans* and *Chlorobaculum tepidum* than in *T. takaii* and *D. acetivorans* due to these thermodynamic differences. The observed differences in the operational direction of the TCA cycle in these species in response to high acetyl-CoA influx due to acetate assimilation can, at least in part, be explained by the thermodynamics of the enzymes involved in citrate cleavage/synthesis.

The activity of ACL with respect to citrate cleavage and synthesis is also affected by the electron donor source. Notably, when the acetate-oxidizing chemoorganoheterotroph *D. postgatei* is cultured with acetate as a carbon source and the sole electron donor, the oTCA cycle functions with ACL, even under a high (20%) CO_2_ partial pressure [52, 53]. To operate the rTCA cycle, reduced ferredoxin (Fd_red_) is required for the conversion of succinyl-CoA to 2-OG by 2-OG:ferredoxin oxidoreductase (OGOR) [14] and for acetyl-CoA conversion to pyruvate by pyruvate:ferredoxin oxidoreductase (POR) (Fig. 2). The reduction of acetyl-CoA by POR is also an essential reaction in gluconeogenesis. Although the machinery involved in Fd reduction in the rTCA cycle has not been experimentally proven [15], *H. thermophilus, T. ammonificans*, and *C. tepidum* use hydrogen or sulfur compounds as an electron donor to generate Fd_red_. In contrast, *D. postgatei* obtains Fd_red_ by oxidizing acetate when provided as the sole electron donor. The finding that *D. postgatei* oxidizes 2-OG to succinyl-CoA with OGOR [52] to provide Fd_red_, which may be used for the reduction of acetyl-CoA to provide pyruvate for gluconeogenesis, suggests that acetyl-CoA reduction via POR is primarily regulated by the Fd_red_ supply. Because the flux of acetyl-CoA reduction should be smaller than that of 2-OG oxidation, ACL catalyzes citrate synthesis when acetate is the sole electron donor.

### Possible simultaneous operation of the Wood–Ljungdahl pathway and reductive tricarboxylic acid cycle

If the WL pathway and rTCA cycle are functioning simultaneously for CO_2_ fixation in a cell, acetyl-CoA influx would be increased compared to cells with only one functional pathway [35]. The presence of extracellular acetate also affects acetyl-CoA influx when the acetate is actively incorporated into cells, as this process serves as an analog of acetyl-CoA influx through CO_2_ fixation by either of the pathways. Therefore, the simultaneous operation of the WL pathway and rTCA cycle can be assessed based on comparisons to cells grown chemolithomixotrophically with acetate and chemolithoautotrophically.

In the present study, we could not find any evidence for the simultaneous operation of the WL pathway and rTCA cycle for CO_2_ fixation in the three examined thermophilic and hydrogenotrophic species. Although combined genomic and proteomic analyses previously suggested that WL pathway is used to fix CO_2_ in *T. ammonificans* [32], the present tracer-based metabolomic analyses of *T. ammonificans* cultured under chemolithoautotrophic conditions did not detect labeled metabolites to support the operation of the WL pathway. Furthermore, the observed growth properties together with the tracer-based metabolomic analyses in *T. indicus* grown chemolithomixotrophically with acetate suggest that the contribution of the WL pathway for acetyl-CoA synthesis was suppressed under high acetyl-CoA influx (Table 1, Fig. S4). This response is distinct from that of *H. thermophilus* grown under chemolithomixotrophic conditions, in which the rTCA cycle continues to operate in the reduction direction with ATP-dependent citrate cleavage enzyme(s). Moreover, previous theoretical analysis [35] suggests that increased acetyl-CoA influx of the WL pathway negatively affects the reductive operation of the TCA cycle with CS, and in fact, the TCA cycle operates oxidatively in the presence of acetate in *T. takaii*. Taken together, these findings suggest that acetyl-CoA production through the WL pathway is suppressed if the rTCA cycle with ACL is active in the same cell, whereas the reductive operation of TCA cycle with CS is suppressed by the WL pathway in the same cell.

No extant organism has been found to possess complete gene sets for both the WL pathway and TCA cycle [32]. Recent tracer-based metabolomic analyses of *Methanothermobacter thermautotrophicus*, which grows chemolithoautotrophically using the WL pathway, have revealed the operation of an incomplete rTCA cycle lacking aconitase and isocitrate dehydrogenase for CO_2_ fixation, as was suggested by previous genomic analyses [40, 54]. This type of incomplete rTCA cycle was also predicted based on comparative genomic analysis to function in *Moorella thermoacetica*, which also has a functional WL pathway [55]. These observations also suggest that organisms do not gain an advantage by simultaneously operating the WL pathway and complete rTCA cycle for CO_2_ fixation.

### Effects of acetyl-CoA influx in non-acetogenic chemolithotrophs in hydrothermal ecosystems

The present results suggest that ATP-dependent citrate cleavage is an essential reaction for rTCA cycle activity in geothermal and hydrothermal microbial ecosystems where organic compounds, such as acetate, are available [5]. In contrast, the WL pathway is adversely affected by increased acetyl-CoA influx, and thus, the contribution of the WL pathway for CO_2_ fixation might be overestimated in these ecosystems. On the other hand, the present study has also demonstrated that the carboxylation of acetyl-CoA to pyruvate and pyruvate to OAA are not affected by the influx of acetyl-CoA in chemolithotrophs with a functional rTCA cycle or WL pathway. These findings are consistent with previous observations in a hydrogenotrophic methanogen and heterotrophic hyperthermophile. The hydrogenotrophic methanogen *M. thermautotrophicus* utilizes an incomplete rTCA cycle to synthesize pyruvate, OAA and 2-OG through CO_2_ fixation in the presence of acetate [40, 56]. The hyperthermophilic and heterotrophic bacterium *Thermotoga neapolitana* also synthesizes pyruvate through the carboxylation of acetyl-CoA in the presence of acetate and high CO_2_ partial pressure [57]. Simultaneous assimilation of CO_2_ and organic carbons is also known in a hyperthermophilic and acidophilic archon that lives in terrestrial hot spring [58] although the effect on the organic carbon to the CO_2_ fixation pathway, 3-hydroxypropionate/4-hydroxybutyrate cycle, remains unrevealed. Taking these into consideration, the contribution of CO_2_ fixation for the synthesis of essential building blocks in both chemolithoautotrophs and chemoorganoheterotrophs cannot be ignored in CO_2_-rich hydrothermal and aphotic geothermal microbial ecosystems, even in the presence of organic compounds. Moreover, they also imply that the importance of CO_2_ fixation and the boundary between autotrophs (or mixotrophs) and heterotrophs in aphotic geothermal and deep-sea hydrothermal ecosystems, as well as the early ecosystems on Earth, is more complicated and blurred than previously thought.

## Conclusion

In summary, the presented results together with those from previous reports have revealed that organic acids, particularly acetate, affect the activity of bacterial carbon fixation systems, including the rTCA cycle and WL pathway, which are two of the dominant CO_2_ fixation systems in aphotic hydrothermal and geothermal microbial ecosystems. Although the impact of increased acetyl-CoA influx in the presence of extracellular acetate is limited in the rTCA cycle, which utilizes ATP-dependent citrate cleavage systems for CO_2_ fixation, such influx affects the operational direction of the reversible TCA (roTCA) cycle, which functions with ATP-independent citrate cleavage enzymes (CS) (15, 22). CO_2_ fixation through the WL pathway is also markedly reduced by increased acetyl-CoA influx, and thus, the contribution of the WL pathway in non-acetogenic chemolithotrophs for primary production is potentially overestimated in hydrothermal and aphotic geothermal microbial ecosystems with available organic acids [1].

## Supplementary Material

Supplementary_information_ycaf227

Supplementary_tables_reveised_ycaf227

## Data Availability

The datasets generated during and/or analyzed during the present study are available from the corresponding author on request.
